# Risk factors of long COVID among discharged patients with omicron infection in Changzhi, China: a retrospective cohort study

**DOI:** 10.3389/fpubh.2026.1774108

**Published:** 2026-08-12

**Authors:** Jianzhou Yang, Jingjing Li, Yuan Li, Liping Yuan, Yanjie Lu, Yuxuan Xue, Tong Wang, Xingyun Xu, Yi Zhu, Zhuo’Ao Zhang, Zhen Qin, Guihua Zhuang, Xiaofeng He

**Affiliations:** 1 Department of Epidemiology and Biostatistics, School of Public Health, Xi’an Jiaotong University Health Science Center, Xi'an, Shaanxi, China; 2 School of Public Health, Changzhi Medical College, Changzhi, China; 3 Institute of Evidence-Based Medicine, Heping Hospital Affiliated to Changzhi Medical College, Changzhi, China; 4 Department of Epidemiology, School of Public Health, Southern Medical University, Guangzhou, China; 5 The First Department of Clinical Medicine, Changzhi Medical College, Changzhi, China

**Keywords:** COVID-19, follow-up study, long Covid, omicron variant, post-acute COVID-19 syndrome

## Abstract

**Background:**

Relatively little is known about long COVID (LC) in China, particularly regarding the effects of recent Omicron sub-lineages. Therefore, we systematically assessed the prevalence and risk factors for developing LC.

**Methods:**

This retrospective cohort study included COVID-19 patients who had been hospitalized at two hospitals in Changzhi, China, between December 15, 2022 and April 30, 2024. All patients were interviewed via telephone ≥ 3 months after discharge. The follow-up study was conducted between July 18 and August 20, 2024. Logistic regression models were used to analyze risk factors for developing long COVID.

**Results:**

A total of 2,765 patients (1,407 male [50.9%]; median [IQR] age 70.0 [59.0–78.0] years) were successfully evaluated by telephone; 458 (16.6%) self-reported long COVID symptoms. The most common symptom was fatigue (4.9%), followed by muscle weakness (3.9%), sleep difficulties (3.0%), brain fog (2.8%) and cough (2.5%). Severe COVID-19 patients had a higher risk of LC than non-severe patients (OR = 1.275, 95% CI: 1.036–1.570). In subgroup analyses stratified by follow-up time, overweight patients (OR = 1.564; 95% CI: 1.052–2.326), patients with ≥ 7 acute-phase symptoms (OR = 1.275; 95% CI: 1.064–1.528), and farmer (OR = 1.439, 95% CI: 1.047, 1.976) had a higher risk of LC, whereas individuals who received immunosuppressant therapy had a lower risk (OR = 0.691, 95% CI: 0.504, 0.948). In addition, aged ≥ 50 years (OR = 2.441, 95% CI: 1.175–5.072) and underweight status (OR = 2.183; 95% CI: 1.115–4.273) were associated with a higher risk of muscle weakness.

**Conclusion:**

This study provides valuable insights into LC after discharge, with 16.6% of discharged patients reporting symptoms. It is essential to monitor at-risk individuals according to identified risk factors for public health efforts.

## Introduction

SARS-CoV-2, the causative agent of COVID-19, has evolved into various sub-variants and serotypes (1, 2). Some patients who recover from acute COVID-19 develop sequelae with persistent symptoms lasting months to years, known as long COVID (LC), post COVID-19 condition (PCC), or post COVID syndrome (PCS) (3, 4). LC is defined as the persistence or emergence of new symptoms 3 months after the initial infection, with symptoms lasting at least 2 months and no alternative explanation (5). It affects an estimated 140 million people worldwide (6). Over 100 LC symptoms have been reported, affecting multiple systems (e.g., cardiovascular, neurologic, respiratory, and musculoskeletal) (7). The most frequently reported symptoms are fatigue, post-exertional malaise, headache, and other neurocognitive problems (8, 9). These individuals have a high risk of sustained health damage related to the declines in physical function and health-related quality of life.

Estimates of the incidence or prevalence of the LC vary greatly in previously published follow-up studies, ranging from 10 to 60%, mainly due to heterogeneity in case definitions, assessment tools, time since infection, and study population selection (8–13). Moreover, most current studies on LC come from Europe and the USA, while studies from Asia are relatively scarce. A meta-analysis observed that studies from the South-East Asia region accounted for only 4.4% (14). In China, research on LC is also limited, focusing mainly on hospitalized patients and earlier SARS-CoV-2 variants (15). Using the keywords “long COVID” and “China” in PubMed for papers published on or after November 15, 2025, we found only 10 studies (16–25) that analyzed the prevalence and risk factors of LC after infection with the Omicron variant in China. However, most of those samples came from infections caused by SARS-CoV-2 sub-variants circulating before 2022 (such as BA.1, BA.2, and even earlier sub-variants), which may not reflect the LC situation after the wave caused by sub-variants such as BA.4, BA.5, BF.7, and XBB. Epidemiological data on LC across a broader range of Omicron sub-lineages are lacking. Therefore, further investigation is needed to explore the risk factors of LC after Omicron infection.

In this study, we used a retrospective cohort study to systematically assess the risk factors of LC among COVID-19 survivors with Omicron infection after discharge in Changzhi, China.

## Methods

### Study design and participants

**Figure 1:**
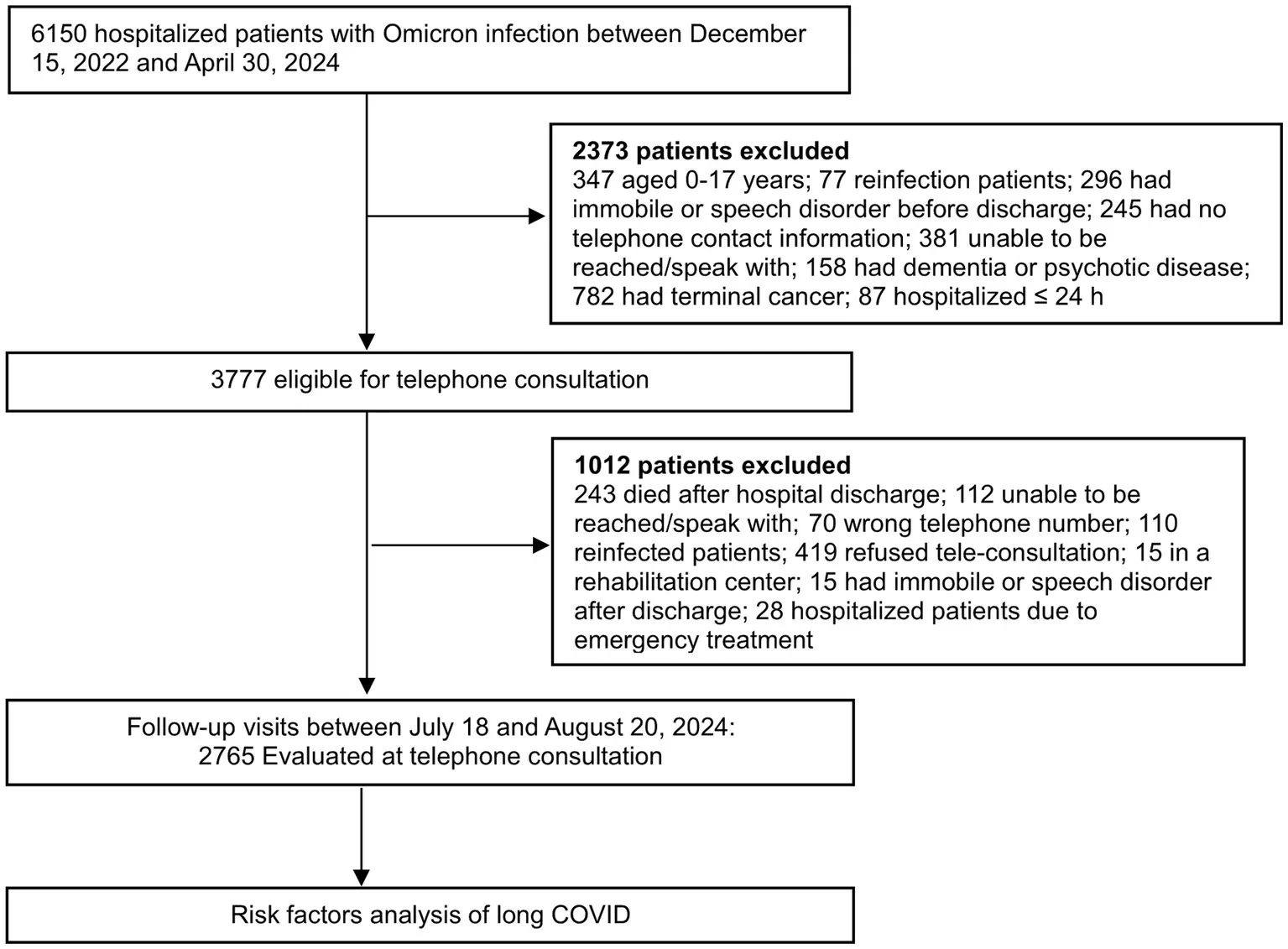
Flow of patient screening and enrollment.

This retrospective cohort study included patients admitted to Heping Hospital Affiliated to Changzhi Medical College and Changzhi People’s Hospital for COVID-19 between December 15, 2022 and April 30, 2024. Inclusion criteria were: (1) age ≥ 18 years, (2) hospitalization for SARS-CoV-2 infection, (3) hospital stay of at least 24 h, and (4) diagnosis of SARS-CoV-2 infection confirmed by RT-PCR, lung computed tomography with clinical features, or both. Exclusion criteria were: (1) death during follow-up after discharge, (2) long-term hospitalization, (3) living in a nursing or welfare home after discharge, (4) terminal cancer, (5) nosocomial SARS-CoV-2 infection, (6) psychotic disorder or dementia, (7) speech disorder, and (8) SARS-CoV-2 reinfection. Based on these criteria, 3,777 COVID-19 discharged patients were eligible and contacted by telephone (Figure 1). In this study, all participants provided verbal informed consent before data collection. The ethics committee of Heping Hospital Affiliated with Changzhi Medical College approved the study (NO. [2024] 087). The Strengthening the Reporting of Observational Studies in Epidemiology (STROBE) reporting guideline was followed.

### Definitions of LC and telephone assessment

LC is defined as conditions occurring in individuals with a history of probable or confirmed SARS-CoV-2 infection, typically developing 3 months from symptom onset, persisting for at least 2 months, and not explained by an alternative diagnosis, according to the World Health Organization (5). We evaluated LC symptoms applying a pre-designed questionnaire (Supplementary Table S1). Discharged patients with one or more of these symptoms were considered to have “LC.”

Patients were contacted by telephone by one infectious disease physician and six trained public health and preventive medicine students between July 18 and August 20, 2024. A structured questionnaire (Supplementary Table S1) was used to investigate LC symptoms. During the call, patients were asked to confirm whether they had any previous or recent illnesses (e.g., influenza or chronic respiratory illnesses) that could cause similar symptoms. The information provided was reviewed before the end of the call to ensure accuracy, and patients were asked for any omissions or additions. After one round of follow-up, 1/10 of the patients were selected for a second follow-up to verify the accuracy of the collected information.

COVID-19 vaccination status was based on self-reported data; most vaccinated individuals reported receiving inactivated COVID-19 vaccine. They were classified as unvaccinated, vaccinated with one dose, two doses, or three doses.

### Data acquisition

We retrospectively collected comprehensive baseline data from hospitalized COVID-19 patients through the hospital’s medical record system, with supplementary information obtained during follow-up to address missing details. The data included demographic and clinical parameters such as age, gender, height, weight, smoking history, occupation, COVID-19 vaccination status, admission and discharge dates, and a range of comorbidities. We also documented the use of antiviral drugs and immunosuppressants, the need for mechanical ventilation, and major symptoms during the acute phase. Severe and non-severe patients were classified according to the guidelines for diagnosis and treatment of SARA-CoV-2 infection (version 10) issued by the National Health Commission of China.

### Statistical analysis

The minimum sample size was estimated as 433 survivors after telephone evaluation, applying the formula N = (Z1-*α*/2/*δ*)2 × p × (1-p) (26), with a 95% confidence interval (CI), a LC prevalence of 8.9% (27), a margin of error of 3% (17), and an anticipated loss to follow-up rate of 20%. Baseline characteristics are presented as counts (percentages, %) for categorical variables and median (interquartile range [IQR]) for continuous variables. Differences in categorical variables were compared using the chi-squared test or Fisher’s exact test, and continuous variables were compared applying the Manne Whitney U test. The crude odds ratio (OR) was derived from the univariate analysis, and the adjusted OR (aOR) from multivariate logistic regression. Variables with *p* < 0.2 in the univariable analysis were included in the multivariable analysis. All tests were two-sided, and *p* < 0.05 was considered statistically significant. Statistical analyses were performed using IBM SPSS Statistics 25.0.

## Results

### Demographic and clinical features of participants

Among 6,150 adult hospitalized patients with Omicron infection between December 15, 2022 and April 30, 2024, 3,777 were eligible and contacted by telephone; and 2,765 (follow-up rate: 73.2%) were successfully evaluated between July 18 and August 20, 2024 after discharge (Table 1; Figure 1). Of these, 1,407 (50.9%) were male, and the median age was 70.0 (IQR: 59.0, 78.0), with 416 (15.1%) aged 18–49 years, 330 (11.9%) aged 50–59 years, 589 (21.3%) aged 60–69 years, 810 (29.3%) aged 70–79 years, and 620 (22.4%) aged ≥ 80 years (Table 1). In total, 1,407 patients (53.1%) were classified as having severe COVID-19. The median (IQR) time from discharge to follow-up was 441 (403–570) days, and the median (IQR) hospital stay was 9 (6–12) days. During hospitalization, 92 patients (3.3%) received mechanical ventilation; 1744 (63.1%) received antiviral treatment and 959 (34.7%) received immunosuppressant therapy. Moreover, 2,607 patients (94.3%) had at least one comorbidity, mainly hypertension (46.9%), cardiovascular disease (32.9%), diabetes (23.3%; Table 1). Furthermore, 760 patients (27.5%) were unvaccinated or had received only one dose of COVID-19 vaccine, while 2005 (72.5%) had received at least two doses (Table 1). Significant differences (*p* < 0.05) between severe and non-severe COVID-19 patients were found regarding age, gender, occupation, COVID-19 vaccination status, number of comorbidities, number of acute symptoms, percentage of smokers, length of hospital stay, antiviral treatment, and immunosuppressant treatment (Table 1).

**Table 1:** Demographic and clinical characteristics of participants.

Characteristics ^a^	Severe disease (*N* = 1,467 [53.1])	Non-severe disease (*N* = 1,298 [46.9])	Overall (*N* = 2,765)	*p* value^**b**^
Age (years)				
Overall (median, IQR)	73.0 (64.0, 81.0)	65.0 (49.8, 75.0)	70.0 (59.0, 78.0)	< 0.001
18–49	92 (6.3)	324 (24.9)	416 (15.1)	< 0.001
50–59	159 (10.8)	171 (13.2)	330 (11.9)	
60–69	311 (21.2)	278 (21.4)	589 (21.3)	
70–79	478 (32.6)	332 (25.6)	810 (29.3)	
≥ 80	427 (29.1)	193 (14.9)	620 (22.4)	
Gender				
Male	853 (58.1)	554 (42.7)	1,407 (50.9)	< 0.001
Female	614 (41.9)	744 (57.3)	1,358 (49.1)	
Occupation				
Farmer	517 (35.2)	352 (27.1)	869 (31.4)	< 0.001
Non-farmer	950 (64.8)	946 (72.9)	1896 (68.6)	
Vaccination				
Unvaccinated or one dose	447 (30.5)	313 (24.1)	760 (27.5)	< 0.001
Two or more doses	1,020 (69.5)	985 (75.9)	2005 (72.5)	
Number of comorbidities				
0	15 (1.0)	143 (11.0)	158 (5.7)	< 0.001
≥ 1	1,452 (99.0)	1,155 (89.0)	2,607 (94.3)	
Comorbidities				
Hypertension	762 (51.9)	536 (41.3)	1,298 (46.9)	< 0.001
Diabetes	342 (23.3)	301 (23.2)	643 (23.3)	0.939
Cardiovascular disease	542 (36.9)	367 (28.3)	909 (32.9)	< 0.001
Cerebrovascular disease	298 (20.3)	241 (18.6)	539 (19.5)	0.247
Respiratory disease	108 (7.4)	435 (33.5)	543 (19.6)	< 0.001
Chronic kidney disease	292 (19.9)	222 (17.1)	514 (18.6)	0.059
Neurodegenerative disorder	23 (1.6)	70 (5.4)	93 (3.4)	< 0.001
Liver disease	315 (21.5)	270 (20.8)	585 (21.2)	0.666
Autoimmune diseases	9 (0.6)	11 (0.8)	20 (0.7)	0.469
Thyroid dysfunction	44 (3.0)	90 (6.9)	134 (4.8)	< 0.001
BMI (kg/m2)				
< 18.5	104 (7.1)	67 (5.2)	171 (6.2)	0.065
18.5–23.9	579 (39.5)	528 (40.7)	1,107 (40.0)	
24–27.9	578 (39.4)	491 (37.8)	1,069 (38.7)	
≥ 28	206 (14.0)	212 (16.3)	418 (15.1)	
Smoke				
Never smoke	1,096 (74.7)	1,035 (79.7)	2,131 (77.1)	0.002
Current/Previous smoke	371 (25.3)	263 (20.3)	634 (22.9)	
Number of acute symptoms				
≤ 2	402 (27.4)	516 (39.8)	918 (33.2)	< 0.001
3–4	660 (45.0)	479 (36.9)	1,139 (41.2)	
5–6	278 (19.0)	205 (15.8)	483 (17.5)	
≥ 7	127 (8.6)	98 (7.5)	223 (8.1)	
Antiviral treatment				
No	416 (28.4)	605 (46.6)	1,021 (36.9)	< 0.001
Yes	1,051 (71.6)	693 (53.4)	1744 (63.1)	
Immunosuppressant therapy				
No	815 (55.6)	991 (76.3)	1806 (65.3)	< 0.001
Yes	652 (44.4)	307 (23.7)	959 (34.7)	
Mechanical ventilation				
No	1,375 (93.7)	1,298 (100.0)	2,673 (96.7)	< 0.001
Yes	92 (6.3)	0 (0.0)	92 (3.3)	
Hospitalization (days)	10.0 (7.0, 14.0)	8.0 (5.0, 11.0)	9.0 (6.0, 12.0)	< 0.001
Follow-up (days)	438 (401, 568)	443 (406, 574)	441 (403, 570)	0.464
90–359	288 (19.6)	279 (21.5)	567 (20.5)	0.468
360–539	568 (38.7)	496 (38.2)	1,064 (38.5)	
≥ 540	611 (41.7)	523 (40.3)	1,134 (41.0)	

IQR, Interquartile range.

a Continuous variables were expressed as medians (interquartile ranges), and categorical variables were expressed as frequencies (percentages).

b *p* values were calculated using rank sum test or chi-square tests. Bold *p* value show statistically significant, i.e., *p* < 0.05.

### Characteristics of LC symptoms during follow-up

**Figure 2:**
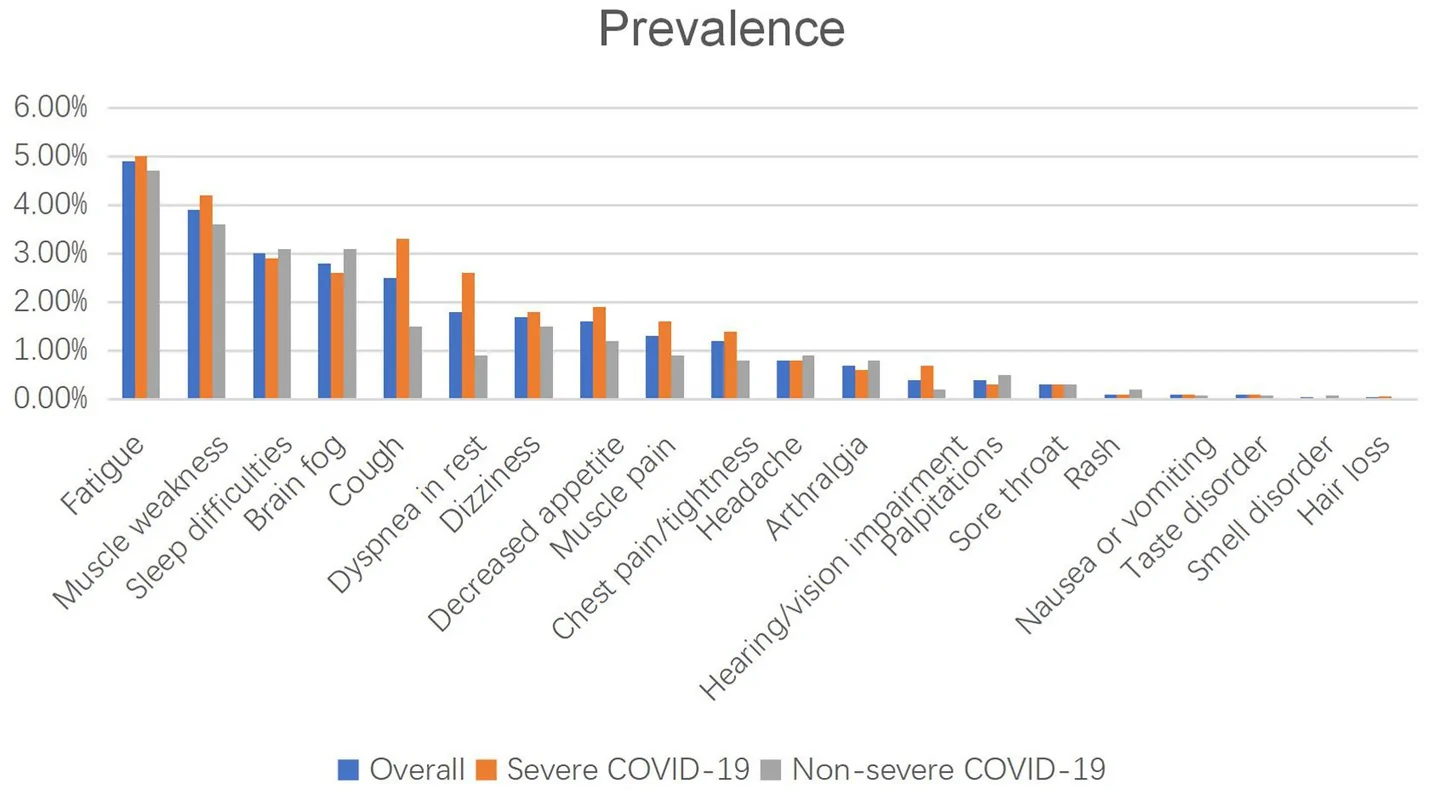
Prevalence of self-reported Long COVID symptoms among COVID-19 discharged patients caused by Omicron variant.

During hospitalization, 2,700 patients (97.7%) had at least one COVID-19-related acute symptom. During follow-up, 458 (16.6%) self-reported at least one LC symptom (Table 2). The most common symptom was fatigue (4.9%), followed by muscle weakness (3.9%), sleep difficulties (3.0%), brain fog (2.8%), and cough (2.5%; Table 2; Figure 2). Compared with non-severe patients, severe COVID-19 patients more frequently self-reported cough, dyspnea at rest, decreased appetite, muscle pain, chest pain/tightness, and hearing/vision impairment (*p* < 0.05, Table 2).

**Table 2:** Results of the telephone assessment on the symptoms of long COVID in discharged patients caused by Omicron variants.

	Severe COVID-19 (*N* = 1,467)	Non-severe COVID-19 (*N* = 1,298)	Overall (*N* = 2,765)	*p* value*
Number of self-reported long COVID symptoms				
0	1,203 (82.0)	1,104 (85.1)	2,307 (83.4)	**0.031**
≥ 1	264 (18.0)	194 (14.9)	458 (16.6)	
Self-reported long COVID symptoms				
Fatigue	74 (5.0)	61 (4.7)	135 (4.9)	0.675
Muscle weakness	62 (4.2)	47 (3.6)	109 (3.9)	0.414
Sleep difficulties	42 (2.9)	40 (3.1)	82 (3.0)	0.735
Brain fog	38 (2.6)	40 (3.1)	78 (2.8)	0.848
Cough	49 (3.3)	20 (1.5)	69 (2.5)	**0.002**
Dyspnea in rest	38 (2.6)	11 (0.9)	49 (1.8)	**< 0.001**
Dizziness	27 (1.8)	19 (1.5)	46 (1.7)	0.107
Decreased appetite	28 (1.9)	15 (1.2)	43 (1.6)	**0.016**
Muscle pain	23 (1.6)	12 (0.9)	35 (1.3)	**0.025**
Chest pain/tightness	21 (1.4)	11 (0.8)	32 (1.2)	**0.033**
Headache	11 (0.8)	12 (0.9)	23 (0.8)	0.932
Arthralgia	8 (0.6)	10 (0.8)	18 (0.7)	0.831
Hearing/vision impairment	10 (0.7)	2 (0.2)	12 (0.4)	**0.011**
Palpitations	4 (0.3)	7 (0.5)	11 (0.4)	0.481
Sore throat	4 (0.3)	4 (0.3)	8 (0.3)	1.000
Rash	2 (0.1)	2 (0.2)	4 (0.1)	1.000
Nausea or vomiting	2 (0.1)	1 (0.08)	3 (0.1)	0.603
Taste disorder	2 (0.1)	1 (0.08)	3 (0.1)	0.603
Smell disorder	0 (0.0)	1 (0.08)	1 (0.04)	1.000
Hair loss	1 (0.07)	0 (0.0)	1 (0.04)	0.470

*Severe COVID-19 vs Non-severe COVID-19. Bold *P* value show statistically significant, i.e., *P* < 0.05.

### Prevalence and risk factors for development of LC

Overall, 458 patients (16.6%) self-reported at least one LC symptom ≥ 3 months after discharge (Table 3). The prevalence was 16.9% (96/567) at 3–12 months, 14.4% (153/1064) at 13–17 months, and 18.4% (209/1134) at ≥ 18 months after discharge (Tables 4–6). During overall follow-up, severe COVID-19 patients had a higher LC risk (OR = 1.275, 95% CI: 1.036–1.570, Table 3) than non-severe patients.

**Table 3:** Logistic regression models to evaluate the risk factors of LC in overall follow-up visits.

Characteristics^a^	Total	Non-LC	LC	Prevalence of LC (%, [95% CI])	OR (95% CI)	*p* value	aOR (95% CI)	*p* value
Overall	2,765	2,307	458	16.6 (15.2, 18.0)				
Age (years)								
18–49	416	357	59	14.2 (11.2, 17.9)	1 (ref)		1 (ref)	
≥ 50	2,349	1950	399	17.0 (15.5, 18.6)	1.238 (0.921, 1.664)	0.157	1.148 (0.843, 1.563)	0.381
Gender								
Male	1,407	1,174	233	16.6 (14.7, 18.6)	1 (ref)		1 (ref)	
Female	1,358	1,133	225	16.6 (14.7, 18.6)	1.001 (0.819, 1.223)	0.995	1.016 (0.828, 1.245)	0.882
Occupation								
Non-farmer	1896	1,595	301	15.9 (14.3, 17.6)	1 (ref)	.	1 (ref)	.
Farmer	869	712	157	18.1 (15.7, 20.8)	1.168 (0.945, 1.445)	0.150	1.147 (0.924, 1.420)	0.214
Vaccination								
Unvaccinated or one dose	760	640	120	15.8 (13.4, 18.6)	1 (ref)	.	1 (ref)	.
Two or more doses	2005	1,667	338	16.9 (15.3, 18.6)	1.081 (0.861, 1.357)	0.500	1.092 (0.869, 1.372)	0.450
Number of comorbidities								
0	158	135	23	14.6 (9.9, 20.9)	1 (ref)	.	1 (ref)	.
≥ 1	2,607	2,172	435	16.7 (15.3, 18.2)	1.176 (0.747, 1.851)	0.485	1.053 (0.659, 1.682)	0.828
BMI (kg/m2)								
< 18.5	171	137	34	19.9 (14.6, 26.5)	1.340 (0.890, 2.016)	0.161	1.284 (0.850, 1.938)	0.236
18.5–23.9	1,107	934	173	15.6 (13.6, 17.9)	1 (ref)	.	1 (ref)	.
24–27.9	1,069	886	183	17.1 (15.0, 19.5)	1.115 (0.888, 1.400)	0.347	1.111 (0.885, 1.396)	0.364
≥ 28	418	350	68	16.3 (13.0, 20.1)	1.024 (0.879, 1.194)	0.760	1.025 (0.879, 1.195)	0.755
Smoke								
Never smoker	2,131	1771	360	16.9 (15.4, 18.5)	1 (ref)	.	1 (ref)	.
Current/Previous smoke	634	536	98	15.5 (12.9, 18.5)	0.899 (0.705, 1.147)	0.393	0.892 (0.698, 1.138)	0.357
Number of acute symptoms								
≤ 2	918	778	140	15.3 (13.1, 17.7)	1 (ref)	.	1 (ref)	.
3–4	1,139	954	185	16.2 (14.2, 18.5)	1.078 (0.848, 1.369)	0.540	1.058 (0.830, 1.349)	0.650
5–6	483	395	88	18.2 (15.0, 21.9)	1.113 (0.961, 1.288)	0.153	.	.
≥ 7	225	180	45	20.0 (15.3, 25.7)	1.116 (0.985, 1.264)	0.084	1.114 (0.983, 1.261)	0.090
Severity with progressive COVID-19								
No	1,298	1,104	194	15.0 (13.1, 17.0)	1 (ref)	.	1 (ref)	.
Yes	1,467	1,203	264	18.0 (16.1, 20.1)	1.249 (1.020, 1.529)	0.032	1.275 (1.036, 1.570)	0.022
Antiviral treatment								
No	1,021	858	163	16.0 (13.8, 18.3)	1 (ref)	.	1 (ref)	.
Yes	1744	1,449	295	16.9 (15.2, 18.8)	1.072 (0.869, 1.321)	0.517	1.023 (0.823, 1.271)	0.838
Immunosuppressant therapy								
No	1806	1,498	308	17.1 (15.4, 18.9)	1 (ref)	.	1 (ref)	.
Yes	959	809	150	15.6 (13.5, 18.1)	0.902 (0.729, 1.116)	0.342	0.821 (0.658, 1.024)	0.081
Mechanical ventilation								
No	2,673	2,232	441	16.5 (15.1, 18.0)	1 (ref)	.	1 (ref)	.
Yes	92	75	17	18.5 (11.9, 27.6)	1.147 (0.671, 1.961)	0.616	1.069 (0.620, 1.845)	0.810

a We produced results using univariate and multivariate logistic regression models, with multivariate analyses using forward stepwise regression analysis. Categorical variables were expressed as frequencies (percentages). OR, odds ratio; CI, confidence interval; LC, long COVID.

**Table 4:** Risk factors associated with LC at 3–12 (7.5 [4.5, 10.3]) months follows-up during Omicron period.

Characteristics^a^	Total	Non-LC	LC	Prevalence of LC (%, [95% CI])	OR (95% CI)	*p* value	aOR (95% CI)	*p* value
Overall	567	471	96	16.9 (14.1, 20.2)	.	.	.	.
Age (years)								
18–49	82	70	12	14.6 (8.6, 23.9)	1 (ref)	.	1 (ref)	.
≥ 50	485	401	84	17.3 (14.2, 20.9)	1.201 (0.623, 2.317)	0.584	1.049 (0.525, 2.098)	0.892
Gender								
Male	290	238	52	17.9 (13.9, 22.8)	1 (ref)	.	1 (ref)	.
Female	277	233	44	15.9 (12.1, 20.7)	0.864 (0.557, 1.342)	0.516	0.854 (0.547, 1.333)	0.488
Occupation								
Non-farmer	389	323	66	17.0 (13.6, 21.0)	1 (ref)	.	1 (ref)	.
Farmer	178	148	30	16.9 (12.1, 23.0)	0.992 (0.618, 1.592)	0.978	0.948 (0.587, 1.531)	0.827
Vaccination								
Unvaccinated or one dose	180	156	24	13.3 (9.1, 19.1)	1 (ref)	.	1 (ref)	.
Two or more doses	387	315	72	18.6 (15.0, 22.8)	1.486 (0.901, 2.450)	0.121	1.545 (0.932, 2.561)	0.091
Number of comorbidities								
0	27	24	3	11.1 (3.9, 28.1)	1 (ref)	.	1 (ref)	.
≥ 1	540	447	93	17.2 (14.3, 20.6)	1.664 (0.491, 5.642)	0.413	1.549 (0.448, 5.364)	0.490
BMI (kg/m2)								
< 18.5	39	30	9	23.1 (12.7, 38.3)	1.370 (0.608, 3.086)	0.448	1.314 (0.580, 2.976)	0.512
18.5–23.9	245	201	44	18.0 (13.7, 23.3)	1 (ref)	.	1 (ref)	.
24–27.9	209	176	33	15.8 (11.5, 21.4)	0.857 (0.522, 1.405)	0.539	0.844 (0.514, 1.387)	0.504
≥ 28	74	64	10	13.5 (7.5, 23.1)	0.845 (0.583, 1.224)	0.373	.	.
Smoke								
Never smoker	394	326	68	17.3 (13.9, 21.3)	1 (ref)	.	1 (ref)	.
Current/Previous smoke	173	145	28	16.2 (11.4, 22.4)	0.926 (0.572, 1.499)	0.754	0.902 (0.554, 1.468)	0.678
Number of acute symptoms								
≤ 2	233	198	35	15.0 (11.0, 20.2)	1 (ref)	.	1 (ref)	.
3–4	214	173	41	19.2 (14.5, 25.0)	1.341 (0.817, 2.200)	0.246	1.241 (0.743, 2.072)	0.409
5–6	82	66	16	19.5 (12.4, 29.4)	1.171 (0.845, 1.624)	0.344	.	.
≥ 7	38	34	4	10.5 (4.2, 24.1)	0.873 (0.606, 1.258)	0.467	0.905 (0.625, 1.310)	0.598
Severity with progressive COVID-19								
No	279	237	42	15.1 (11.3, 19.7)	1 (ref)	.	1 (ref)	.
Yes	288	234	54	18.8 (14.7, 23.7)	1.302 (0.837, 2.026)	0.241	1.290 (0.818, 2.033)	0.274
Antiviral treatment								
No	193	167	26	13.5 (9.4, 19.0)	1 (ref)	.	1 (ref)	.
Yes	374	304	70	18.7 (15.1, 23.0)	1.479 (0.908, 2.410)	0.116	1.531 (0.936, 2.504)	0.089
Immunosuppressant therapy								
No	401	333	68	17.0 (13.6, 20.9)	1 (ref)	.	1 (ref)	.
Yes	166	138	28	16.9 (11.9, 23.3)	0.994 (0.613, 1.610)	0.979	0.956 (0.583, 1.569)	0.859
Mechanical ventilation								
No	548	453	95	17.3 (14.4, 20.7)	1 (ref)	.	1 (ref)	.
Yes	19	18	1	5.3 (0.9, 24.6)	0.265 (0.035, 2.009)	0.199	0.247 (0.032, 1.889)	0.178

^a^We produced results using univariate and multivariate logistic regression models, with multivariate analyses using forward stepwise regression analysis. Categorical variables were expressed as frequencies (percentages). OR, odds ratio, CI, confidence interval, LC, long COVID.

**Table 5:** Risk factors associated with LC at 13–17 (14.3 [13.9, 14.7]) months follows-up during Omicron period.

Characteristics	Total	Non-LC	LC	Prevalence of LC (%, [95% CI])	OR (95% CI)	*p* value	aOR (95% CI)	*p* value
Overall	1,064	911	153	14.4 (12.4, 16.6)				
Age (years)								
18–49	139	122	17	12.2 (7.8, 18.7)	1 (ref)		1 (ref)	
≥ 50	925	789	136	14.7 (12.6, 17.1)	1.237 (0.722, 2.121)	0.439	1.017 (0.579, 1.789)	0.952
Gender								
Male	542	463	79	14.6 (11.9, 17.8)	1 (ref)		1 (ref)	
Female	522	448	74	14.2 (11.5, 17.4)	0.968 (0.687, 1.364)	0.853	1.044 (0.731, 1.491)	0.812
Occupation								
Non-farmer	704	603	101	14.4 (12.0, 17.1)	1 (ref)	.	1 (ref)	.
Farmer	360	308	52	14.4 (11.2, 18.5)	1.009 (0.702, 1.447)	0.966	1.003 (0.694, 1.451)	0.986
Vaccination								
Unvaccinated or one dose	256	218	38	14.8 (11.0, 19.7)	1 (ref)	.	1 (ref)	.
Two or more doses	808	693	115	14.2 (12.0, 16.8)	0.952 (0.640, 1.416)	0.808	0.913 (0.610, 1.368)	0.660
Number of comorbidities								
0	47	42	5	10.6 (4.6, 22.6)	1 (ref)	.	1 (ref)	.
≥ 1	1,017	869	148	14.6 (12.5, 16.9)	1.431 (0.557, 3.675)	0.457	1.168 (0.447, 3.054)	0.752
BMI (kg/m2)								
< 18.5	67	58	9	13.4 (7.2, 23.6)	1.199 (0.559, 2.577)	0.641	1.166 (0.540, 2.513)	0.696
18.5–23.9	419	371	48	11.5 (8.8, 14.9)	1 (ref)	.	1 (ref)	.
24–27.9	423	353	70	16.6 (13.3, 20.4)	1.533 (1.032, 2.275)	0.034	1.564 (1.052, 2.326)	0.027
≥ 28	155	129	26	16.8 (11.7, 23.4)	1.248 (0.964, 1.617)	0.093	1.264 (0.969, 1.647)	0.084
Smoke								
Never smoker	800	680	120	15.0 (12.7, 17.6)	1 (ref)	.	1 (ref)	.
Current/Previous smoke	264	231	33	12.5 (9.0, 17.0)	0.810 (0.536, 1.204)	0.316	0.783 (0.515, 1.191)	0.254
Number of acute symptoms								
≤ 2	327	286	41	12.5 (9.4, 16.6)	1 (ref)	.	1 (ref)	.
3–4	423	369	54	12.8 (9.9, 16.3)	1.021 (0.661, 1.576)	0.926	0.990 (0.639, 1.534)	0.963
5–6	194	163	31	16.0 (11.5, 21.8)	1.152 (0.895, 1.482)	0.272		
≥ 7	120	93	27	22.5 (16.0, 30.8)	1.265 (1.057, 1.514)	0.010	1.275 (1.064, 1.528)	0.009
Severity with progressive COVID-19								
No	496	439	57	11.5 (9.0, 14.6)	1 (ref)		1 (ref)	
Yes	568	472	96	16.9 (14.0, 20.2)	1.566 (1.101, 2.229)	0.013	1.522 (1.068, 2.169)	0.020
Antiviral treatment								
No	312	271	41	13.1 (9.8, 17.3)	1 (ref)	.	1 (ref)	.
Yes	752	640	112	14.9 (12.5, 17.6)	1.157 (0.787, 1.700)	0.459	1.013 (0.683, 1.502)	0.949
Immunosuppressant therapy								
No	740	638	102	13.8 (11.5, 16.5)	1 (ref)	.	1 (ref)	.
Yes	324	273	51	15.7 (12.2, 20.1)	1.168 (0.811, 1.683)	0.403	0.971 (0.662, 1.424)	0.880
Mechanical ventilation								
No	1,023	881	142	11.5 (9.0, 14.6)	1 (ref)	.	1 (ref)	.
Yes	41	30	11	16.9 (14.0, 20.2)	2.275 (1.115, 4.642)	0.024	2.001 (0.962, 4.164)	0.063

Categorical variables were expressed as frequencies (percentages). OR, odds ratio; CI, confidence interval; LC, long COVID.

**Table 6:** Risk factors associated with LC at ≥ 18 (19.1 [18.9, 19.4]) months follows-up during Omicron period.

Characteristics^a^	Total	Non-LC	LC	Prevalence of LC (%, [95% CI])	OR (95% CI)	*p* value	aOR (95% CI)	*p* value
Overall	1,134	925	209	18.4 (16.3, 20.8)				
Age (years)								
18–49	196	166	30	15.3 (10.9, 21.0)	1 (ref)		1 (ref)	
≥ 50	938	759	179	19.1 (16.7, 21.7)	1.305 (0.856, 1.989)	0.216	1.382 (0.894, 2.135)	0.146
Gender								
Male	575	473	102	17.7 (14.8, 21.1)	1 (ref)		1 (ref)	
Female	559	452	107	19.1 (16.1, 22.6)	1.098 (0.813, 1.482)	0.543	1.012 (0.745, 1.373)	0.941
Occupation								
Non-farmer	803	669	134	16.7 (14.3, 19.4)	1 (ref)	.	1 (ref)	.
Farmer	331	256	75	22.7 (18.5, 27.5)	1.462 (1.065, 2.008)	0.019	1.439 (1.047, 1.976)	0.025
Vaccination								
Unvaccinated or one dose	324	266	58	17.9 (14.1, 22.4)	1 (ref)	.	1 (ref)	.
Two or more doses	810	659	151	18.6 (16.1, 21.5)	1.051 (0.752, 1.468)	0.771	1.015 (0.724, 1.423)	0.932
Number of comorbidities								
0	84	69	15	17.9 (11.1, 27.4)	1 (ref)	.	1 (ref)	.
≥ 1	1,050	856	194	18.5 (16.3, 20.9)	1.043 (0.584, 1.861)	0.888	1.099 (0.606, 1.993)	0.755
BMI (kg/m2)								
< 18.5	65	49	16	24.6 (15.8, 36.3)	1.460 (0.790, 2.695)	0.227		
18.5–23.9	443	362	81	18.3 (15.0, 22.2)	1 (ref)		1 (ref)	
24–27.9	437	357	80	18.3 (15.0, 22.2)	1.001 (0.712, 1.410)	0.993		
≥ 28	189	157	22	11.6 (7.8, 17.0)	0.954 (0.762, 1.195)	0.684		
Smoke								
Never smoker	937	765	172	18.4 (16.0, 21.0)	1 (ref)	.	1 (ref)	.
Current/Previous smoke	197	160	37	18.8 (13.9, 24.8)	1.029 (0.694, 1.525)	0.889	1.102 (0.740, 1.642)	0.631
Number of acute symptoms								
≤ 2	358	294	64	17.9 (14.3, 22.2)	1 (ref)	.	1 (ref)	.
3–4	502	412	90	17.9 (14.8, 21.5)	1.003 (0.705, 1.429)	0.985	1.077 (0.753, 1.542)	0.685
5–6	207	166	41	19.8 (15.0, 25.8)	1.065 (0.857, 1.325)	0.570		
≥ 7	67	53	14	20.9 (12.9, 32.1)	1.067 (0.859, 1.324)	0.558	1.079 (0.868, 1.342)	0.491
Severity with progressive COVID-19								
No	523	428	95	18.2 (15.1, 21.7)	1 (ref)		1 (ref)	
Yes	611	497	114	18.7 (15.8, 21.9)	1.033 (0.764, 1.397)	0.831	1.127 (0.823, 1.543)	0.455
Antiviral treatment								
No	516	420	96	18.6 (15.5, 22.2)	1 (ref)		1 (ref)	
Yes	618	505	113	18.3 (15.4, 21.5)	0.979 (0.724, 1.323)	0.890	1.077 (0.787, 1.473)	0.643
Immunosuppressant therapy								
No	665	527	138	20.8 (17.8, 24.0)	1 (ref)		1 (ref)	
Yes	469	398	71	15.1 (12.2, 18.7)	0.681 (0.497, 0.933)	0.017	0.691 (0.504, 0.948)	0.022
Mechanical ventilation								
No	1,102	898	204	18.5 (16.3, 20.9)	1 (ref)		1 (ref)	
Yes	32	27	5	15.6 (6.9, 31.8)	0.815 (0.310, 2.142)	0.679	0.859 (0.325, 2.272)	0.760

a We produced results using univariate and multivariate logistic regression models, with multivariate analyses using forward stepwise regression analysis. Categorical variables were expressed as frequencies (percentages). OR, odds ratio; CI, confidence interval; LC, long COVID.

In subgroup analyses for the 13–17 months period, overweight patients had a higher risk of LC (OR = 1.564; 95% CI: 1.052–2.326, Table 5) compared with patients of normal BMI. Severe COVID-19 patients also had a higher risk (OR = 1.522; 95% CI: 1.068–2.169, Table 5). Patients with ≥ 7 acute-phase symptoms had a higher risk than those with ≤ 2 acute symptoms (OR = 1.275; 95% CI: 1.064–1.528, Table 5).

For the ≥ 18 months period, farmers had a higher risk of LC (OR = 1.439, 95% CI: 1.047–1.976, Table 6), whereas individuals who received immunosuppressant therapy had a lower risk (OR = 0.691, 95% CI: 0.504–0.948, Table 6) of LC. No significant risk factors were identified for the 3–12 months period (Table 4).

### Risk factors for individual LC symptoms

The association between risk factors and the 3 most common symptoms (fatigue, muscle weakness, and sleep difficulties) in LC patients are presented in S2-4 Tables. In this study, we did not find any risk factor significantly associated with developing fatigue and sleep difficulties (Supplementary Tables S2, S4). Compared with patients aged 18–49 years, individuals aged ≥ 50 years was associated with a higher risk of reporting muscle weakness (OR = 2.441, [95% CI: 1.175, 5.072], Supplementary Table S3). Moreover, compared with patients of normal range of BMI, underweight COVID-19 discharged patients had a higher risk of developing muscle weakness (OR = 2.183; 95% CI: 1.115–4.273, Supplementary Table S3).

## Discussion

In this retrospective cohort study, we assessed the prevalence, symptoms, and risk factors of LC among hospitalized patients with Omicron infection after discharge in Changzhi, China. The overall prevalence of self-reported LC was 16.6%. Moreover, the prevalence was 16.9% (96/567) at 3–12 months, 14.4% (153/1064) at 13–17 months, and 18.4% (209/1134) at ≥ 18 months after discharge. This is lower than the prevalence reported for the original (wild-type) strain in China, which ranged from 19.8 to 68.0% at various follow-up times (16, 27–38). The lower prevalence in this study is consistent with the understanding that Omicron infections generally cause less severe acute illness than earlier variants (39), which may reduce the risk of LC. However, our prevalence is slightly higher than the 5.6 to 9.4% reported in some Omicron studies from other Chinese regions (16, 17, 19). These discrepancies likely reflect differences in follow-up duration, patient age (our cohort was older, median 70 years), hospitalization rates (all our patients were hospitalized), and local sub-lineages (e.g., BA.5, BF.7, XBB). Thus, our findings provide new evidence for the post-Omicron LC burden in a hospitalized, older Chinese population. The most common symptoms in our study were fatigue, muscle weakness, sleep difficulties, brain fog, and cough. This pattern broadly aligns with previous Omicron studies (16–18), though some differences exist – for example, hair loss was not prominent in our cohort. These variations may be due to differences in follow-up time, age distribution, and cultural or reporting biases.

Regarding risk factors, we found severe COVID-19 was associated with higher LC risk, consistent with previous studies (27, 38). Severe COVID-19 can lead to pulmonary fibrosis, coagulation dysfunction, virus persistence, and multi-organ damage, all of which require prolonged recovery, and increase vulnerability to new symptoms (40–43). Overweight COVID-19 patients also had higher risk, likely because both obesity and LC involve a proinflammatory state that perpetuates symptoms (44, 45). A high number of acute-phase symptoms (≥ 7) increased LC risk, which aligns with the concept that greater acute symptom burden reflects more severe illness or a more pronounced immune response (46, 47). Interestingly, farmer had a higher LC risk ≥ 18 months after discharge. Farmers constituted a substantial occupational group in our rural study setting. They often have physically demanding work, limited access to continuous healthcare, and may return to heavy labor before full recovery, which could exacerbate or prolong symptoms (48, 49). This finding highlights occupational health disparities that have received little attention in LC research. Immunosuppressant therapy was associated with a lower risk of LC. This supports the hypothesis that an exaggerated immune response during the acute phase contributes to LC. By dampening hyperinflammation (e.g., via IL-6 or IL-23suppression), immunosuppressants may reduce subsequent persistent symptoms (50, 51). However, this should be interpreted cautiously, as the timing and indication of immunosuppressant use (e.g., severe cases with cytokine storm) could introduce confounding. For muscle weakness specifically, age ≥ 50 years and underweight status were significant risk factors. Older adults are prone to sarcopenia and deconditioning after hospitalization. Underweight patients may have poor nutritional reserves, impaired muscle recovery. These findings suggest that nutritional support and early rehabilitation should be prioritized for older and underweight survivors. We did not observe a protective effect of COVID-19 vaccination against LC, unlike some previous reports (14). This may be due to the long interval between vaccination and follow-up (most patients had been vaccinated more than a year earlier), waning immunity, or the predominant use of inactivated vaccines in China, which differ from mRNA vaccines. Further research is needed to be conducted.

These findings highlight several key public health priorities for post-COVID care. Specifically, patients with severe acute illness, overweight status, a high burden of acute-phase symptoms (≥7), and those working as farmers face a significantly higher risk of LC, while individuals receiving immunosuppressant therapy paradoxically show lower risk, suggesting an overactive immune response may contribute to persistent symptoms. Additionally, aged ≥ 50 years and underweight is associated with a markedly increased risk of muscle weakness. Therefore, public health strategies should prioritize targeted surveillance and rehabilitation resources for these high-risk groups, integrate LC prevention into acute-phase management, and address occupational and nutritional disparities to reduce the long-term sequelae of COVID-19.

Several limitations should be considered. First, the study included only hospitalized patients from Changzhi, which may limit generalizability to non-hospitalized individuals or other populations. Second, LC symptoms were self-reported via telephone, without objective validation, which may lead to over- or under- reporting. Third, we did not have a control group of uninfected individuals, making it difficult to attribute all symptoms solely to SARS-CoV-2 infection. Fourth, we lacked biomarker data during the acute phase and follow-up, therefore, we could not explore mechanistic pathways. Fifth, recall bias may have affected reporting of vaccination history. Despite these limitations, this study provides valuable real-world data on LC after Omicron infection in a large cohort of hospitalized Chinese patients.

In summary, 16.6% of discharged COVID-19 patients with Omicron infection reported LC symptoms. Severe COVID-19, overweight, high acute symptom burden, farming occupation, older age, and underweight status were associated with increased risk, while immunosuppressant therapy was associated with lower risk. Monitoring of at-risk individuals and targeted rehabilitation strategies are essential.

## Data Availability

The raw data supporting the conclusions of this article will be made available by the authors, without undue reservation.
