# Supplementary material for: Risk factors of long COVID among discharged patients with omicron infection in Changzhi, China: a retrospective cohort study
Source: Front Public Health. 2026 Aug 12;14:1774108. doi: 10.3389/fpubh.2026.1774108 (PMC13506924; doi:10.3389/fpubh.2026.1774108)
Supplement: Supplementary file 1 [file Presentation_1.ZIP › S1-4 Tables/S2 Table.docx]

**S2 Table. Logistic Regression Models to Evaluate the Risk Factors of Fatigue in overall follow-up visits**

| **Characteristics ^a^** | **Total** | **Non-fatigue** | **Fatigue** | **Prevalence of Fatigue**  **(%, [95% CI])** | **OR (95% CI)** | **P value** | **aOR (95% CI)** | **P value** |
| --- | --- | --- | --- | --- | --- | --- | --- | --- |
| **Overall** | 2765 | 2630 | 135 | 4.9 (4.1, 5.8) | .. | .. | .. | .. |
| **Age (years)** | | | | | | | | |
| 18-49 | 417 | 390 | 27 | 6.5 (4.5, 9.3) | 1 (ref) | .. | 1 (ref) | .. |
| ≥ 50 | 2348 | 2240 | 108 | 4.6 (3.8, 5.5) | 0.696 (0.451, 1.076) | 0.103 | 0.704 (0.452, 1.097) | 0.121 |
| **Gender** | | | | | | | | |
| Male | 1407 | 1338 | 69 | 4.9 (3.9, 6.2) | 1 (ref) | .. | 1 (ref) | .. |
| Female | 1358 | 1292 | 66 | 4.9 (3.8, 6.1) | 0.991 (0.701, 1.400) | 0.957 | 0.912 (0.640, 1.300) | 0.609 |
| **Occupation** | | | | | | | | |
| Non-farmer | 1896 | 1801 | 95 | 5.0 (4.1, 6.1) | 1 (ref) | .. | 1 (ref) | .. |
| Farmer | 869 | 829 | 40 | 4.6 (3.4, 6.2) | 0.915 (0.627, 1.335) | 0.644 | 0.941 (0.641, 1.381) | 1.560 |
| **Vaccination** | | | | | | | | |
| Unvaccinated or one dose | 760 | 720 | 40 | 5.3 (3.9, 7.1) | 1 (ref) | .. | 1 (ref) | .. |
| Two or more doses | 2005 | 1910 | 95 | 4.7 (3.9, 5.8) | 0.895 (0.613, 1.308) | 0.568 | 0.856 (0.584, 1.254) | 0.424 |
| **Number of comorbidities** | | | | | | | | |
| 0 | 158 | 149 | 9 | 5.7 (3.0, 10.5) | 1 (ref) | .. | 1 (ref) | .. |
| ≥ 1 | 2607 | 2481 | 126 | 4.8 (4.1, 5.7) | 0.841 (0.419, 1.687) | 0.625 | 1.086 (0.504, 2.338) | 0.834 |
| **BMI (kg/m2)** | | | | | | | | |
| < 18.5 | 171 | 162 | 9 | 5.3 (2.8, 9.7) | 1.105 (0.535, 2.283) | 0.788 | .. | .. |
| 18.5-23.9 | 1107 | 1054 | 53 | 4.8 (3.7, 6.2) | 1 (ref) | .. | 1 (ref) | .. |
| 24-27.9 | 1069 | 1020 | 49 | 4.6 (3.5, 6.0) | 0.955 (0.642, 1.422) | 0.822 | .. | .. |
| ≥ 28 | 418 | 394 | 24 | 5.7 (3.9, 8.4) | 1.101 (0.859, 1.410) | 0.449 | 1.105 (0.862, 1.417) | 0.432 |
| **Smoke** | | | | | | | | |
| Never smoker | 2131 | 2023 | 108 | 5.1 (4.2, 6.1) | 1 (ref) | .. | 1 (ref) | .. |
| Current/Previous smoke | 634 | 607 | 27 | 4.3 (2.9, 6.1) | 0.833 (0.541, 1.283) | 0.407 | 0.868 (0.562, 1.339) | 0.522 |
| **Number of acute symptoms** | | | | | | | | |
| ≤ 2 | 918 | 880 | 38 | 4.1 (3.0, 5.6) | 1 (ref) | .. | 1 (ref) | .. |
| 3-4 | 1139 | 1076 | 63 | 5.5 (4.4, 7.0) | 1.356 (0.898, 2.048) | 0.148 | .. | .. |
| 5-6 | 483 | 464 | 19 | 3.9 (2.5, 6.1) | 0.974 (0.735, 1.290) | 0.974 | .. | .. |
| ≥ 7 | 225 | 210 | 15 | 6.7 (4.1, 10.7) | 1.183 (0.963, 1.452) | 0.109 | 1.219 (0.989, 1.502) | 0.064 |
| **Severity with progressive COVID-19** | | | | | | | | |
| No | 1298 | 1237 | 61 | 4.7 (3.7, 6.0) | 1 (ref) | .. | 1 (ref) | .. |
| Yes | 1467 | 1393 | 74 | 5.0 (4.0, 6.3) | 1.077 (0.761, 1.525) | 0.675 | 1.229 (0.848, 1.783) | 0.276 |
| **Antiviral treatment** | | | | | | | | |
| No | 1021 | 966 | 55 | 5.4 (4.2, 7.0) | 1 (ref) | .. | 1 (ref) | .. |
| Yes | 1744 | 1664 | 80 | 4.6 (3.7, 5.7) | 0.844 (0.594, 1.201) | 0.347 | 0.906 (0.622, 1.319) | 0.607 |
| **Immunosuppressant therapy** | | | | | | | | |
| No | 1806 | 1710 | 96 | 5.3 (4.4, 6.5) | 1 (ref) | .. | 1 (ref) | .. |
| Yes | 959 | 920 | 39 | 4.1 (3.0, 5.5) | 0.755 (0.516, 1.105) | 0.148 | 0.753 (0.510, 1.112) | 0.154 |
| **Mechanical ventilation** | | | | | | | | |
| No | 2673 | 2543 | 130 | 4.9 (4.1, 5.7) | 1 (ref) | .. | 1 (ref) | .. |
| Yes | 92 | 87 | 5 | 5.4 (2.3, 12.1) | 1.124 (0.449, 2.817) | 0.803 | 1.249 (0.496, 3.146) | 0.637 |

^a^ We produced results using univariate and multivariate logistic regression models, with multivariate analyses using forward stepwise regression analysis. Categorical variables were expressed as frequencies (percentages). OR: odds ratio, CI: confidence interval
