# Supplementary material for: Risk factors of long COVID among discharged patients with omicron infection in Changzhi, China: a retrospective cohort study
Source: Front Public Health. 2026 Aug 12;14:1774108. doi: 10.3389/fpubh.2026.1774108 (PMC13506924; doi:10.3389/fpubh.2026.1774108)
Supplement: Supplementary file 1 [file Presentation_1.ZIP › S1-4 Tables/S3 Table.docx]

**S3 Table. Logistic Regression Models to Evaluate the Risk Factors of Muscle weakness in overall follow-up visits**

| **Characteristics ^a^** | **Total** | **Non-Muscle weakness** | **Muscle weakness** | **Prevalence of Muscle weakness (%, [95% CI])** | **OR (95% CI)** | **P value** | **aOR (95% CI)** | **P value** |
| --- | --- | --- | --- | --- | --- | --- | --- | --- |
| **Overall** | 2765 | 2656 | 109 | 4.1 (3.4, 4.9) | .. | .. | .. | .. |
| **Age (years)** | | | | | | | | |
| 18-49 | 417 | 409 | 8 | 1.9 (1.0, 3.7) | 1 (ref) | .. | 1 (ref) | .. |
| ≥ 50 | 2348 | 2247 | 101 | 4.3 (3.6, 5.2) | **2.298 (1.110, 4.757)** | 0.025 | **2.441 (1.175, 5.072)** | 0.017 |
| **Gender** | | | | | | | | |
| Male | 1407 | 1347 | 60 | 4.3 (3.3, 5.5) | 1 (ref) | .. | 1 (ref) | .. |
| Female | 1358 | 1309 | 49 | 3.6 (2.7, 4.7) | 0.840 (0.572, 1.235) | 0.376 | 0.896 (0.607, 1.332) | 0.580 |
| **Occupation** | | | | | | | | |
| Non-farmer | 1896 | 1824 | 72 | 3.8 (3.0, 4.8) | 1 (ref) | .. | 1 (ref) | .. |
| Farmer | 869 | 832 | 37 | 4.3 (3.1, 5.8) | 0.888 (0.592, 1.331) | 0.564 | 1.037 (0.689, 1.560) | 0.862 |
| **Vaccination** | | | | | | | | |
| Unvaccinated or one dose | 760 | 735 | 25 | 3.3 (2.2, 4.8) | 1 (ref) | .. | 1 (ref) | .. |
| Two or more doses | 2005 | 1921 | 84 | 4.2 (3.4, 5.2) | 1.286 (0.816, 2.025) | 0.279 | 1.332 (0.844, 2.102) | 0.217 |
| **Number of comorbidities** | | | | | | | | |
| 0 | 158 | 155 | 3 | 1.9 (0.7, 5.4) | 1 (ref) | .. | 1 (ref) | .. |
| ≥ 1 | 2607 | 2501 | 106 | 4.1 (3.4, 4.9) | 2.190 (0.687, 6.978) | 0.185 | 1.484 (0.433, 5.088) | 0.530 |
| **BMI (kg/m2)** | | | | | | | | |
| < 18.5 | 171 | 159 | 12 | 7.0 (4.1, 11.9) | **2.183 (1.115, 4.273)** | 0.023 | .. | .. |
| 18.5-23.9 | 1107 | 1070 | 37 | 3.3 (2.4, 4.6) | 1 (ref) | .. | 1 (ref) | .. |
| 24-27.9 | 1069 | 1030 | 39 | 3.7 (2.7, 5.0) | 1.095 (0.693, 1.731) | 0.698 | .. | .. |
| ≥ 28 | 418 | 397 | 21 | 5.0 (3.3, 7.6) | 1.237 (0.941, 1.626) | 0.128 | .. | .. |
| **Smoke** | | | | | | | | |
| Never smoker | 2131 | 2048 | 83 | 3.9 (3.2, 4.8) | 1 (ref) | .. | 1 (ref) | .. |
| Current/Previous smoke | 634 | 608 | 26 | 4.1 (2.8, 5.9) | 1.055 (0.673, 1.654) | 0.815 | 1.001 (0.638, 1.573) | 0.995 |
| **Number of acute symptoms** | | | | | | | | |
| ≤ 2 | 918 | 887 | 31 | 3.4 (2.4, 4.8) | 1 (ref) | .. | 1 (ref) | .. |
| 3-4 | 1139 | 1090 | 49 | 4.3 (3.3, 5.6) | 1.286 (0.813, 2.034) | 0.282 | 1.244 (0.783, 1.977) | 0.355 |
| 5-6 | 483 | 465 | 18 | 3.7 (2.4, 5.8) | 1.052 (0.783, 1.415) | 0.735 | 1.057 (0.781, 1.431) | 0.720 |
| ≥ 7 | 225 | 214 | 11 | 4.9 (2.8, 8.5) | 1.137 (0.899, 1.438) | 0.283 | 1.126 (0.889, 1.426) | 0.325 |
| **Severity with progressive COVID-19** | | | | | | | | |
| No | 1298 | 1251 | 47 | 3.6 (2.7, 4.8) | 1 (ref) | .. | 1 (ref) | .. |
| Yes | 1467 | 1405 | 62 | 4.2 (3.3, 5.4) | 1.175 (0.798, 1.729) | 0.415 | 1.108 (0.742, 1.654) | 0.616 |
| **Antiviral treatment** | | | | | | | | |
| No | 1021 | 985 | 36 | 3.5 (2.6, 4.9) | 1 (ref) | .. | 1 (ref) | .. |
| Yes | 1744 | 1671 | 73 | 4.2 (3.4, 5.2) | 1.195 (0.796, 1.795) | 0.390 | 1.109 (0.729, 1.688) | 0.628 |
| **Immunosuppressant therapy** | | | | | | | | |
| No | 1806 | 1729 | 77 | 4.3 (3.4, 5.3) | 1 (ref) | .. | 1 (ref) | .. |
| Yes | 959 | 927 | 32 | 3.3 (2.4, 4.7) | 0.775 (0.509, 1.180) | 0.234 | 0.720 (0.472, 1.099) | 0.128 |
| **Mechanical ventilation** | | | | | | | | |
| No | 2673 | 2568 | 105 | 3.9 (3.3, 4.7) | 1 (ref) | .. | 1 (ref) | .. |
| Yes | 92 | 88 | 4 | 4.4 (1.7, 10.7) | 1.112 (0.401, 3.085) | 0.839 | 1.092 (0.392, 3.043) | 0.866 |

^a^ We produced results using univariate and multivariate logistic regression models, with multivariate analyses using forward stepwise regression analysis. Categorical variables were expressed as frequencies (percentages). OR: odds ratio, CI: confidence interval
