# Supplementary material for: Risk factors of long COVID among discharged patients with omicron infection in Changzhi, China: a retrospective cohort study
Source: Front Public Health. 2026 Aug 12;14:1774108. doi: 10.3389/fpubh.2026.1774108 (PMC13506924; doi:10.3389/fpubh.2026.1774108)
Supplement: Supplementary file 1 [file Presentation_1.ZIP › S1-4 Tables/S4 Table.docx]

**S4 Table. Logistic Regression Models to Evaluate the Risk Factors of Sleep difficulties in overall follow-up visits**

| **Characteristics ^a^** | **Total** | **Non-sleep difficulties** | **Sleep difficulties** | **Prevalence of Sleep difficulties (%, [95% CI])** | **OR (95% CI)** | ***P* value** | **aOR (95% CI)** | ***P* value** |
| --- | --- | --- | --- | --- | --- | --- | --- | --- |
| **Overall** | 2765 | 2683 | 82 | 3.0 (2.4, 3.7) | .. | *..* | .. | *..* |
| **Age (years)** | | | | | | | | |
| 18-49 | 417 | 407 | 10 | 2.4 (1.3, 4.4) | 1 (ref) | .. | 1 (ref) | .. |
| ≥ 50 | 2348 | 2276 | 72 | 3.1 (2.5, 3.9) | 1.288 (0.659, 2.516) | 0.460 | .. | .. |
| **Gender** | | | | | | | | |
| Male | 1407 | 1370 | 37 | 2.6 (1.9, 3.6) | 1 (ref) | .. | 1 (ref) | .. |
| Female | 1358 | 1313 | 45 | 3.3 (2.5, 4.4) | 1.269 (0.816, 1.973) | 0.290 | .. | .. |
| **Occupation** | | | | | | | | |
| Non-farmer | 1896 | 1839 | 57 | 3.0 (2.3, 3.9) | 1 (ref) | .. | 1 (ref) | .. |
| Farmer | 869 | 844 | 25 | 2.9 (2.0, 4.2) | 0.956 (0.593, 1.540) | 0.852 | .. | .. |
| **Vaccination** | | | | | | | | |
| Unvaccinated or one dose | 760 | 742 | 18 | 2.4 (1.5, 3.7) | 1 (ref) | .. | 1 (ref) | .. |
| Two or more doses | 2005 | 1941 | 64 | 3.2 (2.5, 4.1) | 1.359 (0.800, 2.309) | 0.256 | .. | .. |
| **Number of comorbidities** | | | | | | | | |
| 0 | 158 | 154 | 4 | 2.5 (1.0, 6.3) | 1 (ref) | .. | 1 (ref) | .. |
| ≥ 1 | 2607 | 2529 | 78 | 3.0 (2.4, 3.7) | 1.187 (0.429, 3.286) | 0.741 | .. | .. |
| **BMI (kg/m2)** | | | | | | | | |
| < 18.5 | 171 | 168 | 3 | 1.8 (0.6, 5.0) | 0.600 (0.182, 1.980) | 0.402 | .. | .. |
| 18.5-23.9 | 1107 | 1075 | 32 | 2.9 (2.1, 4.1) | 1 (ref) | .. | 1 (ref) | .. |
| 24-27.9 | 1069 | 1034 | 35 | 3.3 (2.4, 4.5) | 1.377 (0.759, 2.497) | 0.292 | .. | .. |
| ≥ 28 | 418 | 406 | 12 | 2.9 (1.7, 5.0) | 1.183 (0.773, 1.811) | 0.440 | 1.319 (0.850, 2.045) | 0.216 |
| **Smoke** | | | | | | | | |
| Never smoker | 2131 | 2066 | 65 | 3.1 (2.4, 3.9) | 1 (ref) | .. | 1 (ref) | .. |
| Current/Previous smoke | 634 | 617 | 17 | 2.7 (1.7, 4.3) | 0.876 (0.510, 1.505) | 0.631 | .. | .. |
| **Number of acute symptoms** | | | | | | | | |
| ≤ 2 | 918 | 892 | 26 | 2.8 (1.9, 4.1) | 1 (ref) | .. | 1 (ref) | .. |
| 3-4 | 1139 | 1107 | 32 | 2.8 (2.0, 3.9) | 0.992 (0.587, 1.676) | 0.975 | .. | .. |
| 5-6 | 483 | 467 | 16 | 3.3 (2.1, 5.3) | 1.084 (0.790, 1.488) | 0.617 | .. | .. |
| ≥ 7 | 225 | 217 | 8 | 3.6 (1.8, 6.9) | 1.081 (0.827, 1.415) | 0.568 | .. | .. |
| **Severity with progressive COVID-19** | | | | | | | | |
| No | 1298 | 1258 | 40 | 3.1 (2.3, 4.2) | 1 (ref) | .. | 1 (ref) | .. |
| Yes | 1467 | 1425 | 42 | 2.9 (2.1, 3.8) | 0.927 (0.597, 1.439) | 0.735 | .. | .. |
| **Antiviral treatment** | | | | | | | | |
| No | 1021 | 992 | 29 | 2.8 (2.0, 4.1) | 1 (ref) | .. | 1 (ref) | .. |
| Yes | 1744 | 1691 | 53 | 3.0 (2.3, 4.0) | 1.072 (0.677, 1.697) | 0.766 | .. | .. |
| **Immunosuppressant therapy** | | | | | | | | |
| No | 1806 | 1752 | 54 | 3.0 (2.3, 3.9) | 1 (ref) | .. | 1 (ref) | .. |
| Yes | 959 | 931 | 28 | 2.9 (2.0, 4.2) | 0.976 (0.614, 1.551) | 0.917 | .. | .. |
| **Mechanical ventilation** | | | | | | | | |
| No | 2673 | 2592 | 81 | 3.0 (2.4, 3.8) | 1 (ref) | .. | 1 (ref) | .. |
| Yes | 92 | 91 | 1 | 1.1 (0.2, 5.9) | 0.352 (0.048, 2.555) | 0.302 | .. | .. |

^a^ We produced results using univariate and multivariate logistic regression models, with multivariate analyses using forward stepwise regression analysis. Categorical variables were expressed as frequencies (percentages). OR: odds ratio, CI: confidence interval
